# Navitoclax Enhances the Therapeutic Effects of PLK1 Targeting on Lung Cancer Cells in 2D and 3D Culture Systems

**DOI:** 10.3390/pharmaceutics14061209

**Published:** 2022-06-06

**Authors:** Bárbara Pinto, Pedro Novais, Ana C. Henriques, Juliana Carvalho-Tavares, Patrícia M. A. Silva, Hassan Bousbaa

**Affiliations:** 1 UNIPRO—Oral Pathology and Rehabilitation Research Unit, University Institute of Health Sciences (IUCS), Cooperativa de Ensino Superior Politécnico e Universitário (CESPU), Rua Central de Gandra, 1317, 4585-116 Gandra, Portugal; barbara_fernandes_pinto@hotmail.com (B.P.); pedro.ha.novais@gmail.com (P.N.); a24955@alunos.cespu.pt (A.C.H.); 2 Departamento de Fisiologia e Biofísica, Instituto de Ciências Biológicas, Universidade Federal de Minas Gerais, Belo Horizonte 31270-901, Brazil; julianact@ufmg.br; 3 Faculty of Sciences, University of Porto, Rua do Campo Alegre, s/n, 4169-007 Porto, Portugal; 4 Instituto de Ciências Biomédicas Abel Salazar (ICBAS), University of Porto, 4050-313 Porto, Portugal; 5 TOXRUN—Toxicology Research Unit, University Institute of Health Sciences (IUCS), Cooperativa de Ensino Superior Politécnico e Universitário (CESPU), Rua Central de Gandra, 1317, 4585-116 Gandra, Portugal; 6 Centro Interdisciplinar de Investigação Marinha e Ambiental (CIIMAR), Universidade do Porto, Terminal de Cruzeiros do Porto de Leixões, Av. General Norton de Matos s/n, 4450-208 Matosinhos, Portugal

**Keywords:** PLK1, BI2536, Navitoclax, slippage, antimitotics, cancer therapy, mitosis, apoptosis

## Abstract

The efficacy of antimitotics is limited by slippage, whereby treated cells arrested in mitosis exit mitosis without cell division and, eventually, escape apoptosis, constituting a serious resistance mechanism to antimitotics. Strategies to overcome slippage should potentiate the cancer cell killing activity of these antimitotics. Such strategies should accelerate cell death in mitosis before slippage. Here, we undertook a mechanistic analysis to test whether the apoptosis activator Navitoclax potentiates apoptosis triggered by the antimitotic BI2536, a potent inhibitor of Polo-like kinase 1 (PLK1) with the goal of overcoming slippage. We found that cancer cells in 2D cultures treated with BI2536 alone accumulate in mitosis, but a significant fraction of arrested cells undergo slippage and survive. Remarkably, combining BI2536 with Navitoclax dramatically reduces slippage, shifting the cell fate to accelerated death in mitosis. The results are confirmed in 3D spheroids, a preclinical system that mimics in vivo tumor drug responses. Importantly, in 3D spheroids, the effect of the BI2536/Navitoclax combination requires a lower therapeutic dosage of each drug, underlying its potential to improve the therapeutic index. Our results highlight the relevance of apoptosis potentiators to circumvent slippage associated with antimitotics. The combination of BI2536 with Navitoclax shows in vitro synergy/additive effect, which warrants further clinical research.

## 1. Introduction

Lung cancer is the leading cause of cancer-related mortality worldwide, being one of the major challenges to public health [1]. Non-small cell lung cancer (NSCLC) remains the most common lung cancer type, corresponding to 85% of all lung cancer cases [2]. Most NSCLC patients have advanced disease at diagnosis. Platinum-based chemotherapy doublet, including antimicrotubule agents (e.g., paclitaxel/carboplatin and docetaxel/cisplatin), is the standard of care for first-line treatment of advanced NSCLC [3,4,5]. However, only partial responses were achieved with this approach in 30% to 40% of patients [6]. Thus, there is a strong interest in developing more efficacious and safer therapies, thereby improving health management.

The classic antimitotic agents, known as microtubule-targeting agents (MTAs), have been widely used for cancer therapy and remain one of the most successful approaches [7]. MTAs impair a functional mitotic spindle by binding to β-tubulin subunits, leading to spindle assembly checkpoint (SAC) activation and consequent mitotic arrest, which is expected to result in cancer cell death by apoptosis [8]. The SAC is a surveillance mechanism that ensures correct chromosome segregation by monitoring kinetochore–microtubule attachments and chromosome alignment at the metaphase plate [9,10]. SAC operates through the assembly of the mitotic checkpoint complex (MCC) at unattached kinetochores, which diffuses throughout the cell and inhibits the anaphase-promoting complex/cyclosome (APC/C), a ubiquitin ligase that targets securin and cyclin B for proteasomal degradation, resulting in mitotic arrest [11]. When all chromosomes are correctly attached to microtubules, emanating from opposite spindle poles, and aligned at the cell equator, the SAC is silenced, leading to APC/C activation and mitosis progression [9,10].

Despite the therapeutic success of MTAs against several tumor types, including lung cancer, they face some challenges that compromise their efficacy, namely resistance mechanisms developed by tumor cells and the associated toxicity, mainly neurological, gastrointestinal, and myeloid [12,13]. To overcome these drawbacks, the development of alternative strategies to block cells in mitosis without directly targeting microtubules has been gaining more attention. These new approaches consist of inhibiting proteins that play crucial roles during mitosis, especially kinases and kinesins, through small molecules or small interfering RNAs (siRNA), known as second-generation antimitotics (SGAs) [8,14]. One of the targeted proteins is the Polo-like kinase 1 (PLK1), which participates in several mitotic events, including mitotic entry, spindle assembly, kinetochore–microtubule attachment, SAC signaling, and cytokinesis [15]. Several PLK1 inhibitors have been developed that exhibited promising antitumor activity in preclinical models; however, they failed in human clinical trials as monotherapy, stressing the need for the development of strategies to improve their efficacy [14,16,17].

Tumor cells display many cell fate variations after exposure to antimitotic agents, including PLK1 inhibitors [18,19]. Consequently, tumor cells arrested in mitosis could die in mitosis by apoptosis, undergo unequal cell division generating aneuploid daughter cells, or undertake slippage whereby cells exit mitosis without dividing. Slippage occurs due to the slow and gradual degradation of cyclin B even when SAC is on, triggering mitotic exit. What dictates whether the cell dies in mitosis or undergoes slippage is the rate of cyclin B degradation versus the accumulation rate of the apoptotic signal [20]. According to these two competitive network models, if cyclin B levels reach the mitotic exit threshold before the levels of death signals reach the threshold to trigger apoptosis, slippage occurs. Conversely, if death signals reach the threshold to trigger cell death before cyclin B levels fall below the threshold that dictates mitotic exit, cells die in mitosis [19]. Slipped cells can follow three different pathways: they can undergo post slippage death, become senescent, or continue dividing, fueling tumor growth [8,19,21]. Therefore, slippage has been pointed out to be a major resistance mechanism to antimitotics [20].

The efficacy of chemotherapeutics that induce mitotic arrest relies on the intrinsic (or mitochondrial) apoptosis response of the treated cancer cells [22]. The Bcl2 family is essential for this intrinsic apoptosis and consists of three groups of proteins, including anti-apoptotic proteins (BCL-2, BCL-W, BCL-XL, and MCL-1), pro-apoptotic proteins (BAX and BAK), and BH3-only proteins (e.g., BAD, BIK, BIM, BID, and NOXA) [22]. BH3 only members inhibit the anti-apoptotic BCL-2-like proteins (pro-survivals), thereby enabling activation of the pro-apoptotic effectors BAX and BAK, necessary for the mitochondrial pathway of apoptosis, through mitochondrial outer membrane permeabilization. BH3 mimetics have been developed as novel anticancer agents and have shown promise in preclinical studies and clinical trials, particularly in patients with lymphoid malignancies [23].

Since antimitotics, including those that target PLK1, rely on apoptosis to kill cancer cells and given the aforementioned two competitive networks that determine whether mitosis-arrested cells will die or undergo slippage, here, we investigated whether the inhibition of the anti-apoptotic BCL-2 family members can enhance cell death during mitotic arrest caused by a PLK1 inhibitor and, thus, identify a relevant strategy to improve the efficacy of PLK1 targeting, when it is combined with targeted apoptosis potentiators. For this purpose, we used BI2536, a highly selective and potent inhibitor of PLK1, and Navitoclax, a BCL-2 family inhibitor with high affinity toward BCL-2 anti-apoptotic proteins, including BCL-2, BCL-W, and BCL-XL [24,25,26,27]. The strategy was tested on cancer cells in 2D cultures and also in a three-dimensional (3D) cancer model used as a preclinical system to mimic physiologic drug responses. The cellular mechanism by which Navitoclax enhances cancer cell killing by the antimitotic BI2536 was also investigated.

## 2. Materials and Methods

### 2.1. Compounds

BI2536, Navitoclax, Volasertib, and ABT-737 (MedChem Express, Shanghai, China) were reconstituted in sterile dimethyl sulfoxide (DMSO, Sigma-Aldrich Co., Ltd., St. Louis, MO, USA) to a stock concentration of 10 mM. To avoid physicochemical changes and to maintain their integrity and activity, the drugs were stored as small volume aliquots at −20 °C. On the day of the experiment, all compounds were diluted in fresh culture medium at desired concentrations.

### 2.2. Cell Lines and Culture Conditions

NCI-H460 (Non-Small Cell Lung Cancer) cell line was obtained from the European Collection of Cell Culture and was grown in RPMI-1640 culture medium (Roswell Park Memorial Institute, Biochrom, Buffalo, NY, USA) supplemented with 5% heat-inactivated fetal bovine serum (FBS, Biochrom, Berlin, Germany). A549 (Human Lung Adenocarcinoma Epithelial Cells) were obtained from American Type Culture Collection, and HPAEpiC (Human Pulmonary Alveolar Epithelial Cells) was obtained from ScienCell Research Laboratories; both cell lines were grown in DMEM medium (Dulbecco’s Modified Eagle’s, Biochrom), supplemented with 10% heat-inactivated FBS and 1% of non-essential amino acids (Sigma-Aldrich Co., Ltd.). Cell lines were cultured in 25 cm^2^ cell culture flasks (VWR) with complete respective growth culture medium and maintained in a humidified incubator at 37 °C with 5% CO_2_ (Hera Cell, Heraeus, Hanau, Germany).

### 2.3. RNA Isolation and Real-Time PCR Analysis

Total RNA was isolated using the PureZOL^TM^ RNA Isolation Reagent (Bio-Rad Laboratories, Inc. Hercules, Hercules, CA, USA) according to the manufacturer’s instructions and quantified through spectrophotometry (NanoDrop 2000, Thermo Scientific, Waltham, MA, USA). cDNA was synthesized using the iScript^TM^ cDNA Synthesis Kit (Bio-Rad, Hercules, CA, USA) according to the supplier’s instructions, and was amplified using iQ^TM^ SYBR Green Supermix Kit (Bio-Rad) on iQ Thermal Cycler (Bio-Rad), according to the following program: initial denaturing step at 95.0 °C for 3 min; 40 cycles at 94.0 °C for 20 s; 54.0 °C for 30 s and 72.0 °C for 30 s. The primers, used at a final concentration of 10 μM, were: PLK1: forward 5′-CCCCTCACAGTCCTCAATAA-3′ and reverse 5′-TGTCCGAATAGTCCACCC-3′; GAPDH: forward 5′-ACAGTCAGCCGCATCTTC-3′ and reverse 5′- GCCCAATACGACCAAATCC-3′; Actin: forward 5′-AATCTGGCACCACACCTTCTA-3′ and reverse 5′-ATAGCACAGCCTGGATAGCAA-3′. Experiments were performed in triplicate, and the data were acquired using CFX ManagerTM Software (version 1.0, Bio-Rad). The results were analyzed according to ΔCT and normalized against Actin and GAPDH expression levels, which were used as control templates. A fold value of mRNA level ≥ or ≤1.5 relative to that of normal cells was considered as over- or underexpression, respectively.

### 2.4. MTT Assay

The cell viability was determined by tetrazolium salt 3-(4, 5-dimethylthiazol-2-yl)-2, 5-diphenyltetrazolium bromide (MTT) assay. Briefly, A549 and NCI-H460 cells were seeded in 96-well plates at a density of 0.05 × 10^6^ cells/well. After 24 h, the culture medium was replaced with fresh medium containing 2-fold serial dilutions of BI2536, or Navitoclax, or Volasertib, or ABT-737 ranging from 0 to 16,000 nM. Then, 48 hours later, the medium was replaced with 200 µL of fresh medium, and then 20 µL of tetrazolium salt MTT (5 mg/mL PBS) was added to each well. Following 4 h of incubation at 37 °C under darkness, the resulting formazan crystals were solubilized in 100 µL of DMSO. The optical density of the solubilized MTT formazan product was measured at 570 nm using a microplate reader (Biotek Synergy 2, Winooski, VT, USA) coupled with Gen5 software (version 1.07.5, Biotek, Winooski, VT, USA). The percentage of cell viability was expressed as a mean ± standard deviation relative to the control group from three independent experiments performed in triplicate. The mean 50% inhibition concentration (IC_50_) values were calculated using GraphPad Prism version 8 (GraphPad software Inc., San Diego, CA, USA). To analyze the combination treatment effects, a dual-drug crosswise concentration matrix was made for each combination, using concentrations ranging from 0 to 250 nM. The results were analyzed by Combenefit Software (version 2.021, Cancer Research UK Cambridge Institute, Cambridge, UK).

### 2.5. Mitotic Index Determination

A total of 9.0 × 10^4^ A549 and NCI-H460 tumor cells were plated in 6-well plates with complete culture medium and allowed to adhere for 24 h. Then, cells were treated with BI2536 (125 nM for A549 and 62.5 nM for NCI-H460) and Navitoclax (62.5 nM for A549 and 125 nM for NCI-H460), alone or in combination, for 24 h. A549 cells were also treated with 62.5 nM of Volasertib and/or 125 nM of ABT-737. Cells treated with 1 μM of Nocodazole (Sigma-Aldrich Co., Ltd.) were used as a positive control. Additionally, DMSO-treated cells, up to 0.25% concentration, were included as compound solvent control. The mitotic index (MI) was determined by cell-rounding under phase-contrast microscopy using a Nikon TE 2000-U microscope (Nikon, Tokyo, Japan), coupled to a DXM1200F digital camera with Nikon ACT-1 software (Melville, NY, USA). At least 3000 cells were counted from random microscope fields. MI (%) was determined as the ratio between the number of mitotic cells and the total number of cells × 100.

### 2.6. Flow Cytometry Analysis for Apoptosis Detection

Cells from 2D cultures were treated as described for mitotic index determination. After 24 h of treatment, A549 floating and adherent cells were collected and processed with the “Annexin V-FITC Apoptosis Detection Kit” (eBioscience), according to the manufacturer’s instructions. Fluorescence was assessed by BD Accuri™ C6 Plus Flow cytometer (BD Biosciences), and data were analyzed with BD Accuri TM C6 Plus software (version 1.0.27.1, San Jose, CA, USA). At least 20,000 events per sample were collected.

To analyze apoptotic cell death in 3D cultures, 48 h after exposure to BI2536 and/or Navitoclax, or to Volasertib and/or ABT-737, alone or in combination, approximately 32 spheroids were collected from a 96-well ultra-low attachment plate and transferred to a 15 mL centrifuge tube. After precipitation of the spheroids, the supernatants were carefully removed and washed with PBS. Then, 200 µL of trypsin (GIBCO, Invitrogen) was added, and the cells were incubated at 37 °C for 25 min to ensure complete dissociation of spheroids into single cells. Finally, 500 µL of culture medium was added, and the cell suspension was centrifuged at 1000 rpm for 4 min and washed with PBS. The samples were treated with “Annexin V-FITC Apoptosis Detection Kit” according to the manufacturer’s instructions. At least 20,000 events per sample were collected.

### 2.7. Death Fluorometric TUNEL Assay

A total of 9.0 × 10^4^ of A549 cells were grown on poly-l-lysine-coated coverslips for 24 h, and cells were treated as described for mitotic index determination. Immediately after the treatment, cells were fixed in 4% paraformaldehyde (*w*/*v*, Sigma-Aldrich Co., Ltd.) in PBS for 10 min. Then, cells were washed in PBS and permeabilized with 0.2% (*v*/*v*) Triton X-100 (Sigma-Aldrich Co., Ltd.) in PBS for 5 min. The DeadEnd Fluorometric TUNEL System (Promega, Madison, WI, USA) was used according to the manufacturer’s instructions. The 4′,6-diamidino-2-phenylindole (DAPI, Sigma-Aldrich Co., Ltd.) was used to stain DNA at 2 μg/mL in Vectashield mounting medium (Vector, Newark, CA, USA). Images were acquired with an Axio Observer Z.1 SD microscope (Carl Zeiss, Oberkochen, Germany), with the Plan Apochromatic 63×/NA 1.4 objective, coupled to an AxioCam MR3. The images were then processed using ImageJ (version 1.51, Rasband, W.S., ImageJ, U.S. National Institutes of Health, Bethesda, MD, USA). TUNEL-positive cells were counted from a total of approximately 500 cells from 10 random fields under a fluorescence microscope. Then, the apoptotic index (the percentage of positively TUNEL-stained cells over the total of cells) was determined.

### 2.8. Live-Cell Imaging

For live-cell imaging experiments, 9.0 × 10^4^ A549 cells were plated onto LabTek II chambered cover glass (Nunc) with complete culture medium and maintained for 24 h at 37 °C with 5% CO_2_. Then, cells were treated with 125 nM of BI2536, or with 62.5 nM of Navitoclax, or with a combination of both compounds. A differential interference contrast (DIC) optics, with a 63× objective on an Axio Observer Z.1 SD inverted microscope, equipped with an incubation chamber with a temperature of 37 °C in a 5% CO_2_ atmosphere, was used to capture images at 5 min intervals up to 48 h. The time-lapse images were used to generate movies using ImageJ software (version 1.51). Cell fate was followed since the first mitosis. Dead cells were classified as death in mitosis (DiM), when cells died in mitosis; as post-mitotic death (PMD), when cells died after complete cell division; or as post slippage death (PSD), when cells died following mitotic exit without cell division. Cells that exited mitosis without dying and survived were classified as post slippage survival (PSS).

### 2.9. 3D Spheroid Formation and Drug Treatment

A549 cells were seeded at 4000 cells/well into the 96-well ultra-low attachment plates (Corning 7007, Fisher Scientific, Pittsburgh, PA, USA) in order to promote the self-assembly of cells into three-dimensional (3D) cellular spheroids. After 4 days, the spheroids were treated with BI2536 and Navitoclax, or with Volasertib and ABT-737, in a dual-drug crosswise concentration matrix, using concentrations ranging from 0 to 16,000 nM. Then, 48 h later, 3D spheroids viability and apoptosis were determined.

### 2.10. CellTiter-Glo Viability Assay

Spheroid viability, based on ATP measurement, was determined by CellTiter-Glo 3D cell viability assay (Promega) according to the manufacturer’s instructions. Briefly, A549 spheroids in 96-well ultra-low attachment culture plate were exposed for 48 h to BI2536 and/or Navitoclax, or to Volasertib and/or ABT-737. Then, they were transferred separately into single wells of a 96-well opaque culture plate (Fisher Scientific), and after exposure to CellTiter-Glo^®^ 3D reagent for 10 min, the luminescence signal was measured using a microplate reader. The results were expressed as the percentage of cell viability relative to the control group. The BI25365, Navitoclax, Volasertib, and ABT-737 IC_50_ values were calculated using GraphPad Prism version 8.

### 2.11. Statistical Analysis

Statistical analysis was performed using one-way ANOVA followed by the Tukey’s post-test or unpaired *t*-test in GraphPad Prism version 8. Experiments were performed in triplicate, and the data expressed as the mean ± standard deviation (SD). *p*-values of * *p* < 0.05, ** *p* < 0.01, *** *p* < 0.001, and **** *p* < 0.0001 defined the level of statistical significance.

## 3. Results

### 3.1. PLK1 Is Upregulated in Lung Cancer Cells

**Figure 1:**
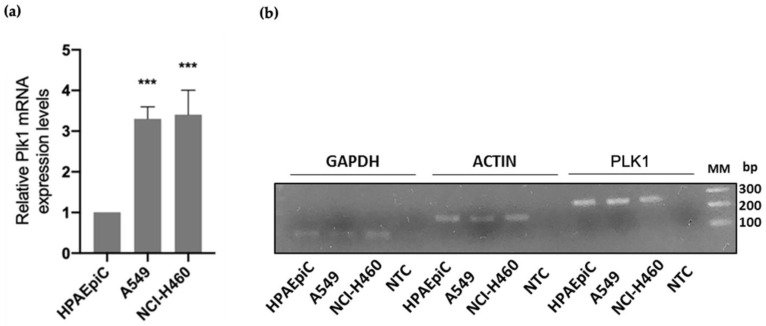
PLK1 is overexpressed in NSCLC cell lines. (**a**) Relative mRNA expression of PLK1 was determined by qRT-PCR in NCI-H460 and A549 cancer cell lines, and compared to non-tumor HPAEpiC cell line, showing PLK1 overexpression in tumor cell lines with statistical relevance of *** *p* < 0.001 by unpaired *t*-test from three independent experiments. (**b**) Representative 1% agarose gel image of PCR products is shown. MM-molecular marker; bp-base pair; NTC-Non-Template Control.

To assess the relevance of PLK1 as a potential target for cancer therapy, we analyzed its expression in A549 and NCI-H460 non-small cell lung cancer (NSCLC) cell lines (Figure 1). Analysis of PLK1 mRNA levels by qRT-PCR demonstrated that PLK1 was overexpressed in both cancer cell lines tested when compared to the non-tumor cell line HPAEpiC. There was a 3.3 ± 0.3 and 3.4 ± 0.6-fold increase in PLK1 expression in A549 and NCI-H460 cell lines, respectively, compared to the lung non-tumor cell line. These results are in concordance with previous studies that reported a PLK1 overexpression in NSCLC, highlighting the relevance of PLK1 targeting [28,29].

### 3.2. Navitoclax Enhances the Antiproliferative Effect of BI2536-Mediated PLK1 Inhibition in Lung Cancer Cells

**Figure 2:**
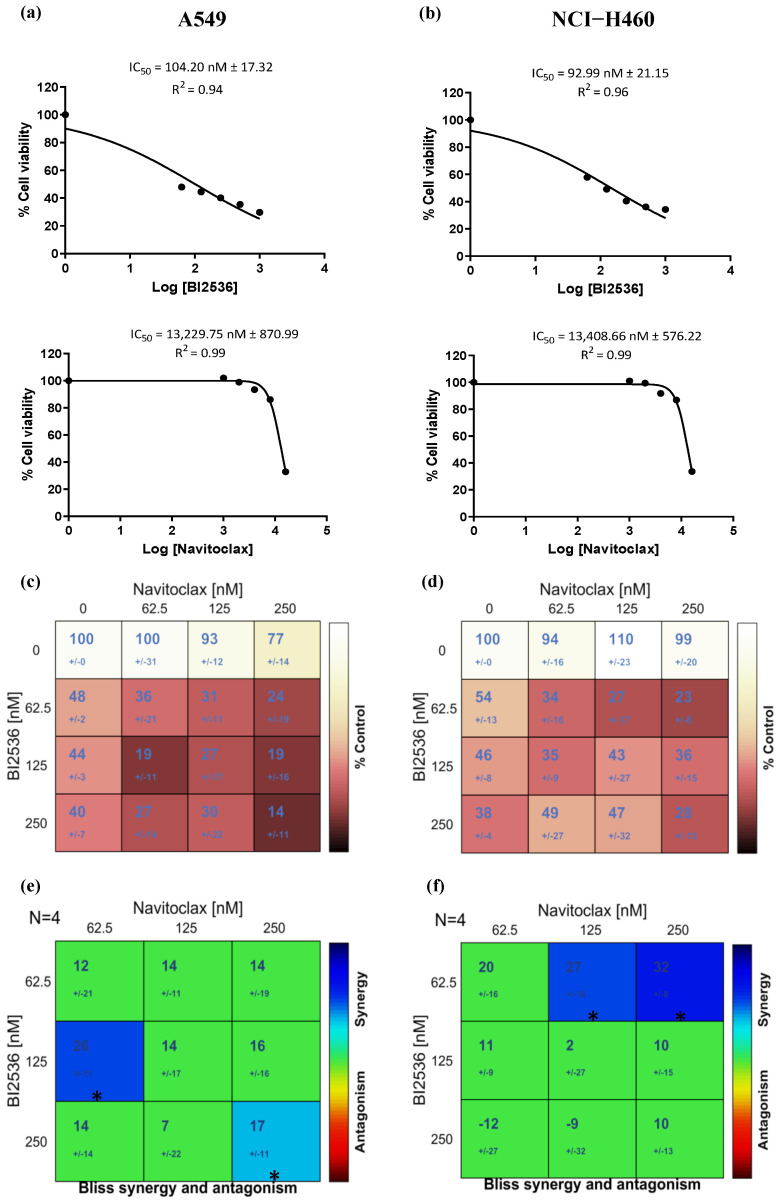
BI2536/Navitoclax combination exacerbates cytotoxicity of lung cancer cells. Dose−response curve of BI2536 and Navitoclax in A549 (**a**) and NCI−H460 (**b**) cell lines. Cell viability (%) of mono or dual−drug combinations after 48 h of treatment in A549 (**c**) and NCI−H460 (**d**) cells, from four independent experiments, as determined by MTT assay. Synergy scores calculated by the Bliss model of Combenefit software with statistical relevance of * *p* < 0.05 in A549 (**e**) and NCI-H460 (**f**).

To explore the antiproliferative effect of PLK1 inhibition in combination with an apoptotic potentiator, we first determined the concentration of the PLK1 inhibitor BI2536 and the BH3-mimetic Navitoclax, able to cause 50% cell viability inhibition (IC_50_), after 48 h exposure, in the two lung cancer cell lines, A549 and NCI-H460. Figure 2a,b summarizes the IC_50_ values of the single agents on both cell lines. BI2536 showed dose-dependent antiproliferative effects on both cell lines, with an IC_50_ of 104.20 ± 17.32 nM and 92.99 ± 21.15 nM for A549 and NCI-H460 cells, respectively (Figure 2c,d). Navitoclax had little antitumor effect on both cancer cell lines, showing cytotoxicity only at concentrations higher than 13,000 nM (Figure 2c,d). Remarkably, in all the BI2536/Navitoclax combinations tested, cell viability was severely affected, and in many cases, the decrease associated with the dual-drug combination was higher than the sum of the decrease induced by each of the matching single-agent treatments (Figure 2c,d). Interestingly, such a potentially synergistic effect was also observed with the combinations where the concentrations of the single agents were low, which is very relevant, from a therapeutic perspective, to minimize toxicity and side effects reported in clinical trials for both drugs [14,30]. Based on these results, synergy matrices were created (Figure 2e,f). The synergy matrix of A549 showed that 125 nM BI2536 was the first concentration where synergism was detected in combination with 62.5 nM Navitoclax. The synergy matrix of NCI-H460 showed that 62.5 nM BI2536 was the first concentration where synergism was detected in combination with 125 nM Navitoclax. We selected these synergy points for subsequent experiments to investigate the cellular mechanistic underlying the enhanced cytotoxicity of the combinations.

### 3.3. Combining BI2536-Mediated PLK1 Inhibition with Navitoclax Overcomes Slippage and Shifts the Cancer Cell Fate to Accelerated Death in Mitosis

**Figure 3:**
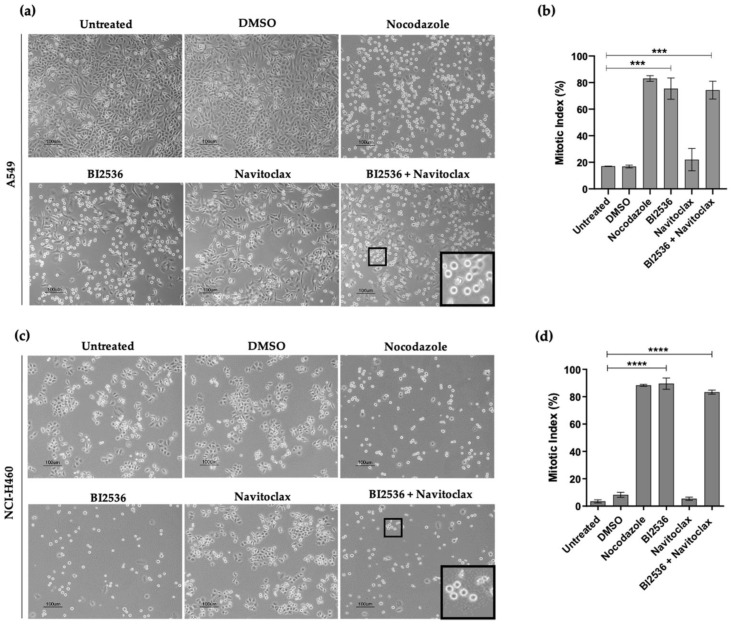
BI2536, but not Navitoclax, induces mitotic arrest of lung cancer cells. Representative phase-contrast microscopy images of untreated BI2536 (125 nM in A549 and 62.5 nM in NCI-H460), and Navitoclax (62.5 nM in A549 and 125 nM in NCI-H460) co-treated cells, for 24 h, showing accumulation of rounded and bright cells (mitotic cells) in A549 (**a**) and NCI-H460 (**c**) cell lines. Cells treated with up to 0.25% DMSO (compound solvent) and 1μM Nocodazole (antimitotic agent) were used as controls. Mitotic index graph showing accumulation of A549 (**b**) and NCI-H460 (**d**) mitotic cells with statistical relevance of *** *p* < 0.001 and **** *p* < 0.0001 by one-way ANOVA with Tukey’s multiple comparisons test from three independent experiments. Bar, 100 µm. Data were expressed as mean ± SD.

In order to unveil the cellular mechanistic associated with the enhanced antiproliferative effect induced by BI2536 in combination with Navitoclax, A549 and NCI-H460 cells were treated for 24 h with the above combinations with single agents or with medium or DMSO (controls) and then examined by phase-contrast microscopy. For BI2536 single treatment, and as expected, we observed an accumulation of bright and round cells reminiscent of cells arrested in mitosis, similarly to Nocodazole, a known antimitotic agent, used here as a positive control (Figure 3a,c). This observation was confirmed by the calculation of the mitotic index (MI), which was significantly increased in both BI2536-treated cancer cell lines (75.4 ± 8.0% and 89.6 ± 4.1% in A549 and NCI-H460, respectively) compared to untreated (17.1 ± 0.2%% and 3.3 ± 1.4% for A549 and NCI-H460 cells, respectively) and DMSO-treated cells (16.9 ± 1.0% and 8.3 ± 1.8% for A549 and NCI-H460 cells, respectively) (Figure 3b,d). Treatment with Navitoclax alone did not affect normal cell cycling, apart from a few dead cells. As mitosis is not supposed to be affected by Navitoclax, the increase in the mitotic index observed in the BI2536/Navitoclax combinations should be attributed to BI2536. This result confirms the previously reported antimitotic activity of BI2536-mediated PLK1 inhibition, thereby validating the use of PLK1 inhibitor in our study for the subsequent studies to unveil the cellular mechanistic of the enhanced cell toxicity in combination with Navitoclax.

**Figure 4:**
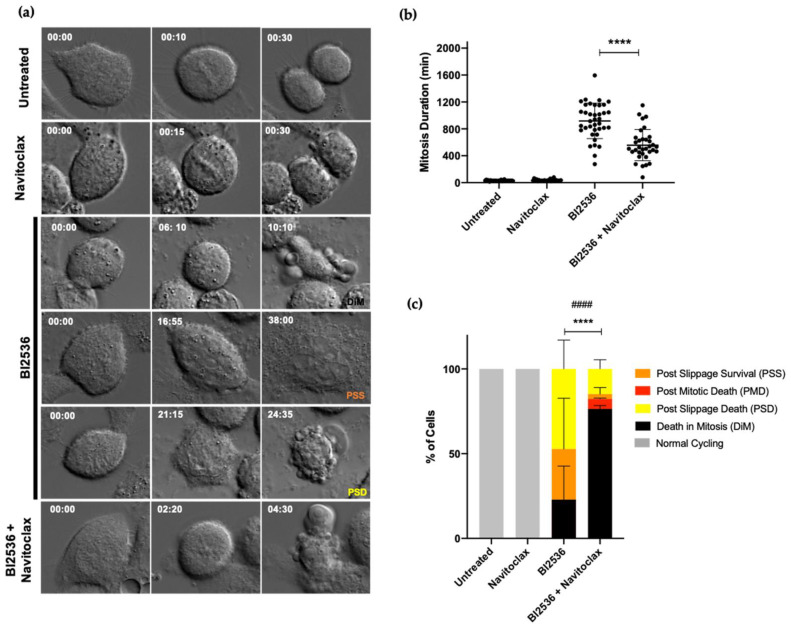
Navitoclax prevents slippage and accelerates cell death in mitosis in lung cancer cells treated with BI2536. (**a**) Representative time-lapse sequences of untreated, BI2536-, Navitoclax- and BI2536/Navitoclax-treated cells. Untreated (*n* = 27) and Navitoclax-treated cells (*n* = 25) undertake mitosis for about 30 min. BI2536-treated cells (*n* = 38) arrest in mitosis during several hours (918.2 ± 263.5 min) and die in mitosis (top) or undergo slippage followed by death (bottom) or remain alive (middle). BI2536/Navitoclax-treated cells (*n* = 34) spend less time in mitosis (558.3 ± 232.0 min) than BI2526-treated cells. Numbers indicate time in 00 h:00 min. (**b**) Quantification of mitosis duration in the different treatments, as determined by time-lapse microscopy, with statistical relevance of **** *p* < 0.0001 by one-way ANOVA with Tukey’s multiple comparisons test. Each dot represents one cell. (**c**) Quantification of cell fate under the indicated treatments. Percentage of cells undergoing death in mitosis (DiM), post-mitotic death (PMD), post slippage death (PSD), cells that remain alive after slippage (post slippage survival, PSS), and cells with normal cycling, with statistical relevance of **** *p* < 0.0001 (DiM) and #### *p* < 0.0001 (Slippage [PSD +PSS]) by two-way ANOVA with Tukey’s multiple comparisons test. The error bars represent mean ± SD of three independent experiments.

Antimitotic agents induce prolonged mitotic arrest through activation of the SAC [31]. Then, treated cells either die in mitotic arrest or slip out of mitosis, without cell division, into an abnormal G1 state in which they may die, arrest in a tetraploid G1 state, or continue to proliferate [19]. Slippage is a major mechanism contributing to drug resistance [20]. Therefore, an ideal strategy should shift the fate of antimitotic-treated cells to death instead of slippage. To this end, we analyzed the cell fates after treatment with the BI2536/Navitoclax combination, taking advantage of time-lapse microscopy, which allows monitoring the spatiotemporal dynamics of live cells (Figure 4a).

A549 tumor cells were treated with BI2536 and Navitoclax, alone or in combination, and each cell was followed over 48 h live-cell time-lapse analysis. As expected, BI2536-treated cells lasted in mitosis for several hours (918.2 ± 263.5 min) (Figure 4b), while untreated and Navitoclax-treated cells undertook mitosis for about 30 min (30.5 ± 7.3 min and 36.0 ± 14.1 min, respectively) (Figure 4b, Video S1 and Video S2, respectively). Interestingly, the addition of Navitoclax to BI2536-treated cells shortened the duration of mitotic arrest (558.2 ± 232.0 min) as compared to cells treated with BI2536 only, suggesting that the time from mitotic arrest to death was shortened (Figure 4b). Concerning cell fates, all Navitoclax-treated cells were able to divide normally, indicating that Navitoclax alone is not toxic to tumor cells, at least at the concentration used (Figure 4c and Video S2). As to BI2536-treated cells, different fates were observed: 22.9 ± 19.8% died in mitosis (DiM), and 77.1 ± 18.8% underwent slippage, of which 64.8 ± 31.3% died after slippage (post slippage death, PSD) and 35.2 ± 31.3% survived (post slippage survival, PSS) during the recorded time (Figure 4c, Video S3, Video S5, and Video S4, respectively). In cells treated with the BI2536/Navitoclax combination, 76.4 ± 2.0% of cells died in mitosis (Figure 4c and Video S6), 5.9 ± 0.5% of cells divided but died in the following interphase (post-mitotic death, PMD), and, remarkably, only 17.7 ± 1.5% underwent slippage (of which 83.3 ± 23.6% PSD and 16.7 ± 23.6% PSS) (Figure 4c). Taken together, the results show that combining BI2536-mediated PLK1 inhibition with an apoptosis potentiator minimizes slippage and, thus, drug resistance by shifting the cell fate to accelerated death in mitosis, likely through accelerating the accumulation of apoptotic signals.

**Figure 5:**
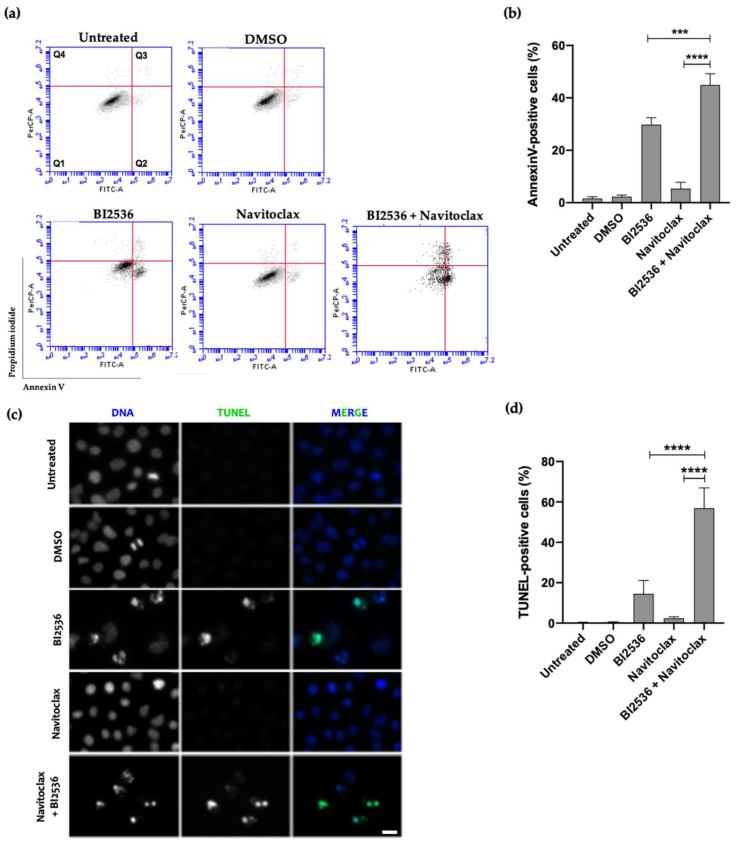
Combination of BI2536 and Navitoclax potentiates cancer cell death by apoptosis. (**a**,**b**) Flow cytometry analysis of apoptosis, in A549 cell line, by Annexin V/PI co-staining, after 48 h treatment. The quadrants Q were defined as Q1 = live (Annexin V- and PI-negative), Q2 = early stage of apoptosis (Annexin V-positive/PI-negative), Q3 = late stage of apoptosis (Annexin V- and PI-positive), and Q4 = necrosis (Annexin V-negative/PI-positive). (**b**,**d**) Quantification of A549 (**b**) Annexin V-positive cells are shown with statistical relevance of *** *p*  <  0.001 and **** *p*  <  0.0001 by one-way ANOVA with Tukey’s multiple comparisons test. (**c**) Representative images of A549 apoptotic cells after 48 hours’ treatment, revealed by TUNEL assay to detect DNA fragmentation (green). DNA (blue) was stained with DAPI. Bar, 5 µm. (**d**) Quantification of A549 TUNEL-positive cells. **** *p* < 0.0001, by one-way ANOVA with Tukey’s multiple comparisons test. The error bars represent mean ± SD of three independent experiments.

Cell death in the BI2536/Navitoclax combination was mainly by apoptosis, as revealed both by Annexin V/PI and TUNEL assays (Figure 5). Indeed, flow cytometry analysis of Annexin V/PI-stained cells showed that the percentage of apoptotic cells in the BI2536/Navitoclax combination was significantly higher than that of BI2536-only treatment (44.9 ± 4.3% vs. 29.8 ± 2.7%, respectively), being residual in untreated, DMSO- or Navitoclax-treated cells (1.6 ± 0.7%, 5.4 ± 2.4%, and 2.3 ± 0.6%, respectively). This result was corroborated by the TUNEL assay (Figure 5c,d).

### 3.4. Co-Treatment with BI2536 and Navitoclax Decrease 3D Cancer Spheroid Viability

**Figure 6:**
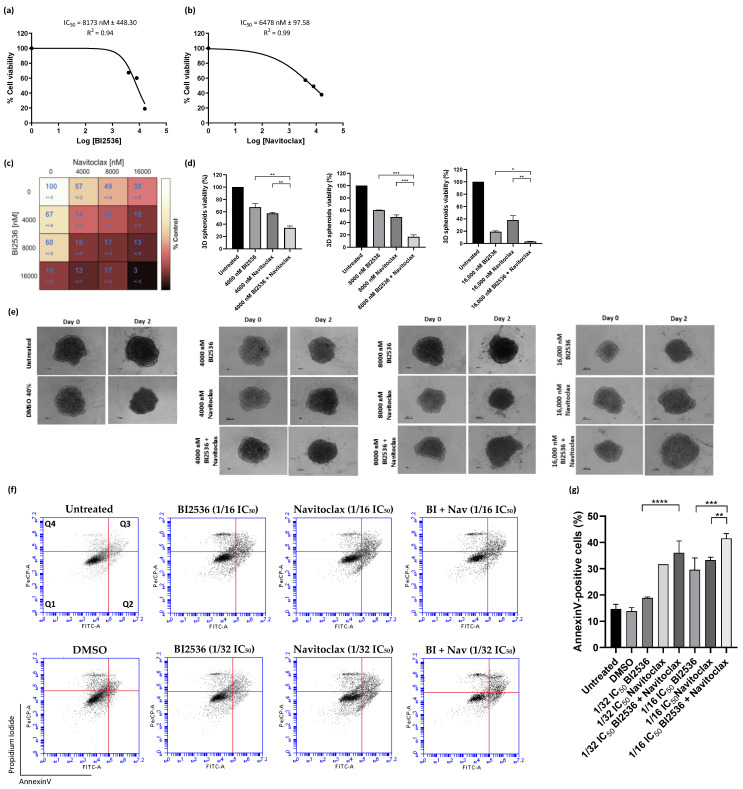
BI2536/Navitoclax combination potentiates 3D spheroid cytotoxicity and cell death. Dose−response curve of BI2536 (**a**) and Navitoclax (**b**) in A549 spheroids. (**c**) Cell viability (%) of single or dual−drug combinations after 48 h of treatment, using the Combenefit software package. (**d**) The combinatory therapy significantly reduced the 3D spheroid viability at the indicated concentrations. (**e**) Representative images of A549 3D spheroids at days 0 and 2 post-treatment with mono− or BI2536/Navitoclax combinations (100 μm). Representative cytograms (**f**) and quantification (**g**) of Annexin V−positive cells are shown for A549 cancer cell line. The quadrants Q were defined as Q1 = live (Annexin V− and PI−negative), Q2 = early stage of apoptosis (Annexin V−positive/PI−negative), Q3 = late stage of apoptosis (Annexin V− and PI−positive), and Q4 = necrosis (Annexin V−negative/PI−positive). Data represent the mean ± SD, one-way ANOVA followed by Tukey’s multiple comparisons test, * *p* < 0.05; ** *p* < 0.01; *** *p* < 0.001 and **** *p* < 0.0001.

Three-dimensional (3D) spheroid cell cultures exhibit several characteristics of in vivo tumor tissues, such as cell–cell interaction, hypoxia, pH gradient, drug penetration, response and resistance, and production/deposition of extracellular matrix [32]. Therefore, the spheroids can be used to closely mimic a solid in vivo tumor. We, thus, established spheroids of A549 cells and evaluated the cytotoxic activity of 48 h BI2536 treatment, individually or in combination with Navitoclax, by CellTiter-Glo assay (Figure 6). Both BI2536 and Navitoclax showed dose-dependent antiproliferative effects on A549 spheroids, with an IC_50_ of 8173.0 ± 448.3 nM and 6478.0 ± 97.58 nM (Figure 6a,b). The BI2536 IC_50_ was much higher than its corresponding IC_50_ in the 2D cultures, probably due to the aforementioned characteristics of the 3D cultures. We also performed a spheroid viability matrix containing 16 different combinations at concentrations of 0, 4000, 8000, and 16,000 nM of BI2536 and Navitoclax (Figure 6c). In all the BI2536/Navitoclax combinations tested, cell viability was severely affected, being the decrease at least equal to the sum of the decrease induced by each of the matching single-agent treatments (Figure 6d). Interestingly, we still observed an additive effect of the BI2536/Navitoclax combination when spheroids were treated with 4000 nM concentration of each agent, a concentration much lower than the respective IC_50_ (33.95 ± 2.3% viability with the combination, 67.4 ± 4.2% with BI2536, and 57.4 ± 1.3% with Navitoclax). From macroscopic observations, the spheroids of A549 cells treated with BI2536 or Navitoclax individual agents were loosely compacted and partially fragmented, with many cells that lost adhesion to the spheroid surface, indicative of cytotoxicity, as compared to the intact control spheroids (Figure 6e). This phenotype was exacerbated after treatment with the BI2536/Navitoclax combinations, leading to even more flattened and loosely compacted spheroids. Annexin V/PI co-staining followed by flow cytometry analysis showed exacerbation of cell death by apoptosis in spheroids treated with BI2536/Navitoclax combinations, even at concentrations as low as 1/32 or 1/16 × IC_50_ of each agent (Figure 6f,g).

Overall, and similarly to the effect on 2D cancer cultures, combining the antimitotic BI2536 with the apoptosis activator Navitoclax potentiates cancer cell death in a model that mimics a solid in vivo tumor. The required cytotoxic concentration of BI2536 is higher for 3D cancer cultures, but a lower therapeutic dosage of each individual drug is required in the combination regimen. Such BI2536/Navitoclax combination therapy may be able to prevent the toxic effects on non-cancer cells while simultaneously producing cytotoxic effects on cancer cells.

## 4. Discussion

Inhibition of PLK1 has been extensively explored as a therapeutic strategy against cancer [33,34,35,36]. Several PLK1 inhibitors entered clinical trials and globally demonstrated poor efficacy [14]. Mitotic slippage has been pointed out as one of the reasons for the therapeutic failure of antimitotic agents, including PLK1 inhibitors [18,20,37]. In this study, we showed that the efficacy of the PLK1 inhibitor BI2536 was significantly improved when combined with Navitoclax, an inhibitor of the anti-apoptotic proteins of the BCL-2 family, as a strategy to overcome slippage and accelerate apoptosis. Similar results were observed when alternative small molecule inhibitors of PLK and BCL-2 family members, namely Volasertib and ABT-737, respectively, were used; therefore, excluding potential off-target effects (Figures S1 and S2).

Since what dictates whether cells die in mitosis or undergo slippage in response to antimitotic agents is the relative rate of cyclin B degradation and the accumulation of death signals, our results suggest that Navitoclax favors the accumulation of death signals in cancer cells arrested in mitosis by BI2536, thereby, accelerating apoptosis in these cells, before slippage occurs. These results are in agreement with previous studies that reported increased cell death when apoptosis was targeted in cells treated with paclitaxel or an inhibitor of kinesin-5 in 2D cultures [38,39,40,41]. Therefore, targeting apoptosis could be a general strategy to increase the antitumor efficacy of antimitotics. This is particularly relevant given that many cancers develop resistance to antimitotics, exhibit deficient SAC or acquire resistance apoptosis [42,43]. For instance, patients whose ovarian tumor tissue expresses high BCL-XL levels are less sensitive to taxane treatment, highlighting further the relevance of the addition of BCL-XL inhibitors, such as Navitoclax, to improve taxane-based therapy [44]. Importantly, we showed that the BI2536/Navitoclax combination enhances tumor cell death by apoptosis at concentrations significantly lower than their respective IC_50_. Thus, the combination provides an opportunity to reduce the dosage of both compounds, which is expected to minimize their toxicity and other side effects. At the same time, this result is particularly relevant to overcoming possible resistance to Navitoclax. Indeed, the anti-apoptotic BCL-2 and BCL-XL are frequently expressed at high levels in a variety of cancers, and also in NCI-H460 and A549, which may contribute to chemoresistance of cancer cells [45,46,47].

Navitoclax, as a single agent, has demonstrated limited outcomes in clinical trials on particular cancer types, namely acute lymphocytic leukemia (ALL) and advanced small cell lung cancer (SCLC), probably due to different expression levels of the BCL-2 family proteins, being thrombocytopenia and neutropenia the most adverse events [48,49]. Compared to its potent antitumor efficacy in preclinical studies, the efficacy of BI2536 as a single agent in clinical studies was moderate, namely in patients with NSCLC, advanced exocrine adenocarcinoma of the pancreas, and different types of lymphoma [14,50,51]. In this context, the synergistic efficacy, at low doses, demonstrated by the BI2536/Navitoclax combination in the present study is encouraging and highlights its potential to overcome the disappointing outcomes and adverse effects reported for both compounds in clinical trials as monotherapy.

3D cell cultures have been widely used due to their capacity to reproduce the in vivo tumor microenvironment, including oxygen and nutrient gradients, cell heterogeneity, gene expression, and cell-to-cell and cell-to-extracellular matrix interactions [32,52]. To validate the findings observed in 2D monolayer cell cultures, 3D models were used as an alternative to recapitulate the real tumor. We showed that the BI2536/Navitoclax combination was also significantly cytotoxic to 3D spheroids, even at lower concentrations of the single agents. This suggests that our strategy to overcome slippage subsequent to antimitotic treatment could be applied to the complex environment of the real tumor, with an efficacy that is similar to that observed in 2D monolayer cultures, presumably with reduced toxicity to non-cancer cells. A previous study also demonstrated that the combination of Navitoclax with Carfilzomib (a proteasome inhibitor) potentiated apoptotic induction in 3D spheroids of pancreatic tumor cells (PANC1) [53].

Our results present, however, some limitations that deserve to be addressed. The 3D spheroid cell cultures used in this study consisted of an avascular system, with only lung tumor cells, while the tumor microenvironment encompasses other cell types such as tumor-associated fibroblasts, immune, and endothelial cells, which affect drug efficacy [32]. Therefore, further research with heterotypic and vascular spheroids, as well as with xenograft models of human lung cancer, is required to validate the synergistic interaction between BI2536 and Navitoclax in an environment that is close to the real in vivo tumor. PLK1 is overexpressed in several cancers, including NSCLC [54]. Notwithstanding, it remains to be elucidated if these cancers are more sensitive to PLK1 inhibition than cancers with normal expression levels of PLK1. In the same line of thought, it remains to be addressed whether the efficacy of the combinational treatment is determined by the expression levels of the anti-apoptotic proteins (BCL-2, BCL-XL, BCL-W, and MCL-1). Answering these questions should clarify whether the expression levels of the anti-apoptotic proteins and PLK1 can be used to help clinicians to select those cancer patients who may benefit from combination treatments.

**Figure 7:**
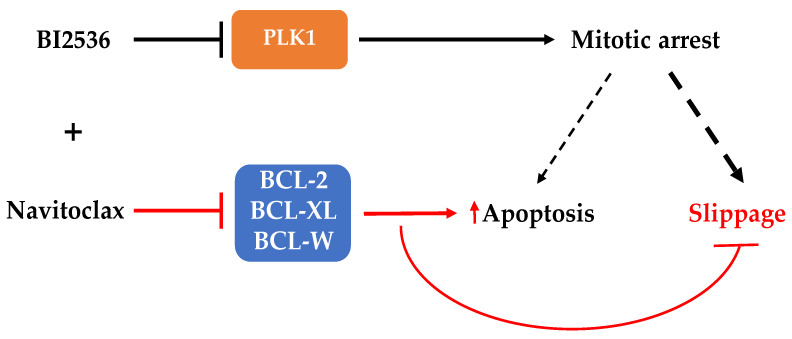
Model for the effects of the combination of PLK1 inhibition and Navitoclax in human lung cancer cells. Cancer cells arrested in mitosis under individual treatment with PLK1 inhibitor BI2536 can die in mitosis by apoptosis, or exit mitosis by slippage, the most frequent cell fate (larger dashed line). The addition of Navitoclax (+), an inhibitor of the indicated BCL-2 family members, can synergistically potentiate BI2536-mediated apoptosis during the mitotic arrest and, thus, prevents mitotic slippage, highlighting the therapeutic potential of targeting PLK1 in combination with an apoptotic inducer. Black and red lines represent the BI2536 and Navitoclax effects, respectively.

In conclusion, we showed that the efficacy of the antimitotic BI2536 could be improved, either in 2D or 3D culture systems, if apoptosis is accelerated by the use of an apoptosis potentiator such as Navitoclax to prevent slippage (Figure 7). This finding is relevant as it gives BI2536, which previously entered clinical trials but unsuccessfully, a second chance to be used to fight cancer, hopefully with successful outcomes.

## Supplementary Materials

The following supporting information can be downloaded at: https://www.mdpi.com/article/10.3390/pharmaceutics14061209/s1. Video S1: Time-lapse imaging (DIC microscopy) of a normal mitosis in untreated A549 cell, time is shown in hours:minutes, available online at https://youtu.be/vlTdWvAr16U. Video S2: Time-lapse imaging (DIC microscopy) of a A549 cell treated with Navitoclax undergoing a normal mitosis, time is shown in hours:minutes, available online at https://youtu.be/zZSR-kfCzfQ. Video S3: Time-lapse imaging (DIC microscopy) of a A549 cell treated with BI2536 undergoing death in mitosis after spending more than 13 h in mitosis, time is shown in hours:minutes, available online at https://youtu.be/EjwSWnHe0_s. Video S4: Time-lapse imaging (DIC microscopy) of a A549 cell treated with BI2536 undergoing slippage and remaining alive, time is shown in hours:minutes, available online at https://youtu.be/EjLqOKQ4cHo. Video S5: Time-lapse imaging (DIC microscopy) of a A549 cell treated with BI2536 undergoing slippage followed by death, time is shown in hours:minutes, available online at https://youtu.be/nARMNfPHCXY. Video S6: Time-lapse imaging (DIC microscopy) of a A549 cell treated with BI2536 and Navitoclax undergoing cell death in mitosis after arresting in mitosis for less than 3 h, time is shown in hours:minutes, available online at https://youtu.be/2q1wI-J47j0. Figure S1: Volasertib/ABT-737 combination exacerbates cytotoxicity of lung cancer cells. Dose-response curve of Volasertib and ABT-737 in A549 cell line (a). Cell viability (%) of mono or dual-drug combinations after 48 hours of treatment in A549 cells (b), from two independent experiments, as determined by MTT assay. Synergy scores calculated by the Bliss model of Combenefit software with statistical relevance of * *p* < 0.05 in A549 cells (c). Volasertib, but not ABT-737, induces mitotic arrest of lung cancer cells. Representative phase contrast microscopy images of untreated, Volasertib (62.5 nM), and ABT-737 (125 nM) co-treated cells, for 24 h, showing accumulation of rounded and bright cells (mitotic cells). Cells treated with up to 0.25% DMSO (compound solvent), and 1 μM Nocodazole (antimitotic agent) were used as controls (d). Mitotic index graph showing accumulation of A549 mitotic cells (e) with statistical relevance of **** *p* < 0.0001 by one-way ANOVA with Tukey’s multiple comparisons test from three independent experiments. Bar, 10 µm. Data were expressed as mean ± SD; Figure S2: Volasertib/ABT-737 combination potentiates 3D spheroid cytotoxicity and cell death. Dose-response curve of Volasertib (a) and ABT-737 (b) in A549 spheroids. (c) Cell viability (%) of single or dual-drug combinations after 48 hours of treatment, using the Combenefit software package. (d) The combinatory therapy reduced significantly the 3D spheroid viability at the indicated concentrations. (e) Representative images of A549 3D spheroids at days 0 and 2 post-treatment with mono- or Volasertib/ABT-737 combinations (100 μm). Representative cytograms (f) and quantification (g) of Annexin V-positive cells are shown for A549 cancer cell line. The quadrants Q were defined as Q1 = live (Annexin V- and PI-negative), Q2 = early stage of apoptosis (Annexin V-positive/PI-negative), Q3 = late stage of apoptosis (Annexin V- and PI-positive) and Q4 = necrosis (Annexin V-negative/PI-positive). Data represent the mean ± SD, One-way ANOVA followed by Tukey’s multiple comparisons test, * *p* < 0.05; *** *p* < 0.001 and **** *p* < 0.0001.
