# Supplementary material for: Navitoclax Enhances the Therapeutic Effects of PLK1 Targeting on Lung Cancer Cells in 2D and 3D Culture Systems
Source: Pharmaceutics. 2022 Jun 6;14(6):1209. doi: 10.3390/pharmaceutics14061209 (PMC9229367; doi:10.3390/pharmaceutics14061209)
Supplement: Supplementary file 1 [file pharmaceutics-14-01209-s001.zip › pharmaceutics-1761228-supplementary.pdf]

Supplementary Materials

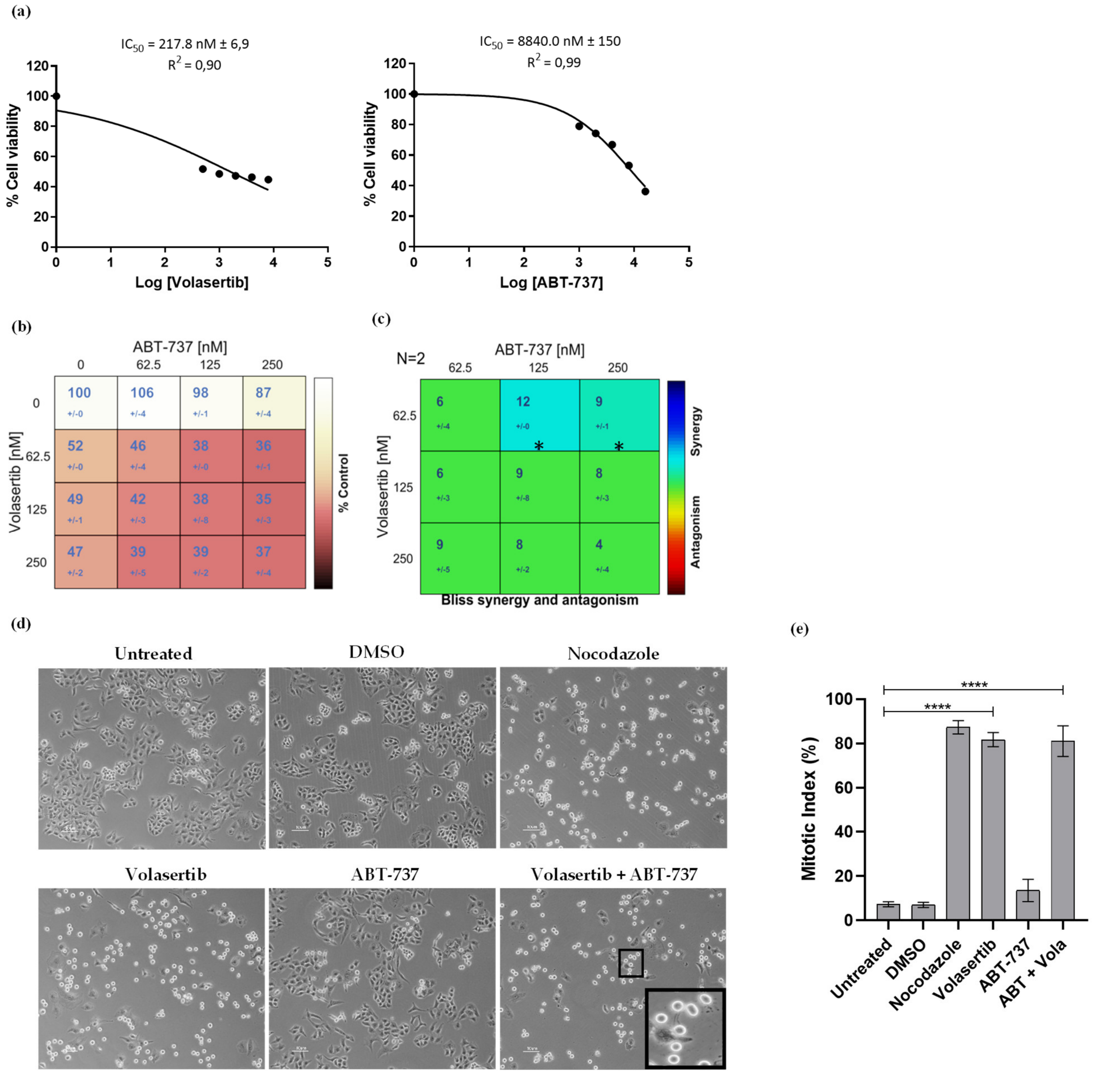

Figure S1. Volasertib/ABT-737 combination exacerbates cytotoxicity of lung cancer cells. Dose-response curve of Volasertib and ABT-737 in A549 cell line (a). Cell viability (%) of mono or dual-drug combinations after 48 hours of treatment in A549 cells (b), from two independent experiments, as determined by MTT assay. Synergy scores calculated by the Bliss model of Combenefit software with statistical relevance of * $p < 0.05$ in A549 cells (c). Volasertib, but not ABT-737, induces mitotic arrest of lung cancer cells. Representative phase contrast microscopy images of untreated, Volasertib (62.5 nM), and ABT-737 (125 nM) co-treated cells, for 24 hours, showing accumulation of rounded and bright cells (mitotic cells). Cells treated with up to 0.25% DMSO (compound solvent), and 1μM Nocodazole (antimitotic agent) were used as controls (d). Mitotic index graph showing accumulation of A549 mitotic cells (e) with statistical relevance of ****$p < 0.0001$ by one-way ANOVA with Tukey's multiple comparisons test from three independent experiments. Bar, 10 μm. Data were expressed as mean ± SD.

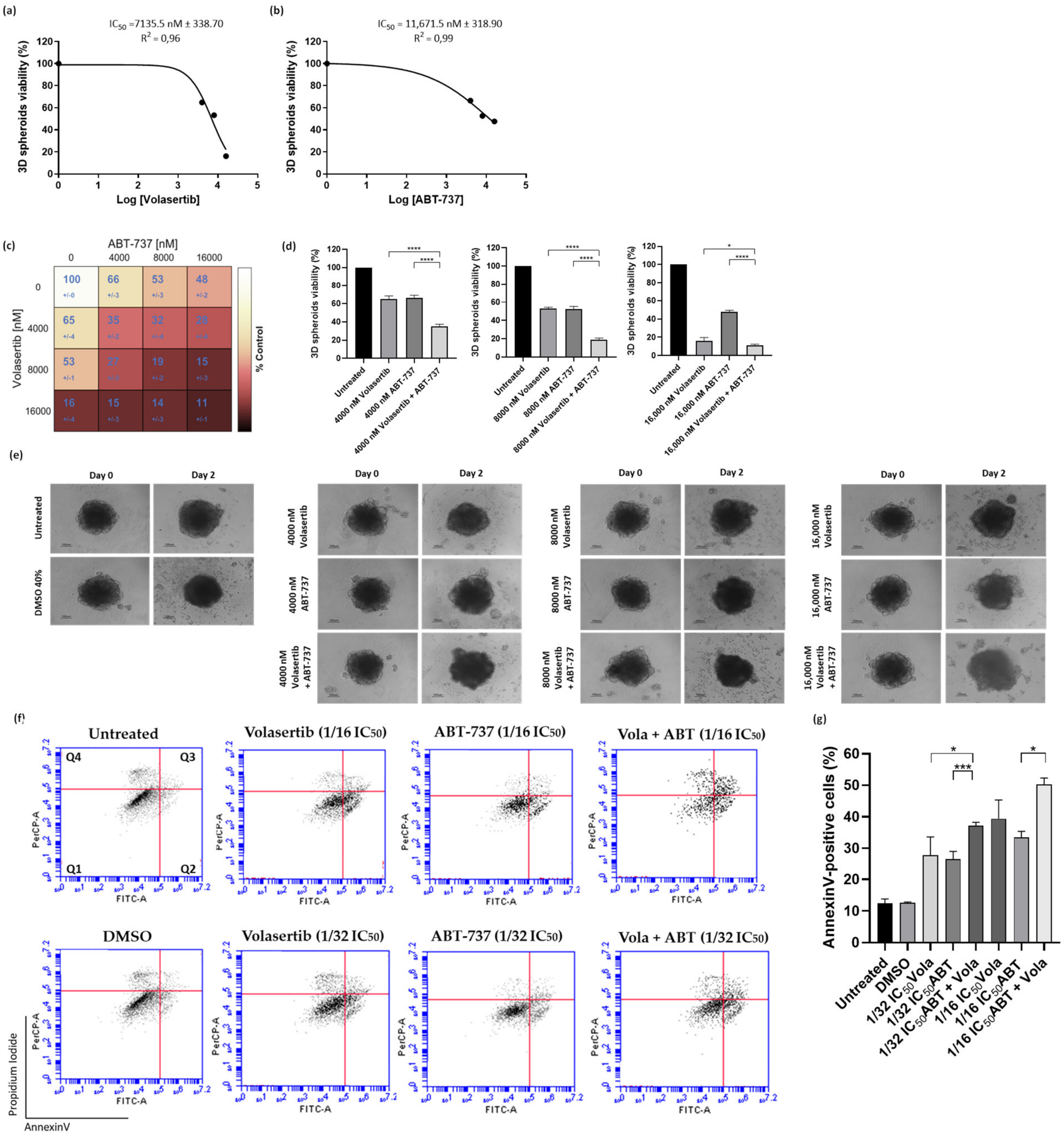

Figure S2: Volasertib/ABT-737 combination potentiates 3D spheroid cytotoxicity and cell death. Dose-response curve of Volasertib (a) and ABT-737 (b) in A549 spheroids. (c) Cell viability (%) of single or dual-drug combinations after 48 hours of treatment, using the Combenefit software package. (d) The combinatory therapy reduced significantly the 3D spheroid viability at the indicated concentrations. (e) Representative images of A549 3D spheroids at days 0 and 2 post-treatment with mono- or Volasertib/ABT-737 combinations (100 µm). Representative cytograms (f) and quantification (g) of Annexin V-positive cells are shown for A549 cancer cell line. The quadrants Q were defined as Q1 = live (Annexin V- and PI-negative), Q2 = early stage of apoptosis (Annexin V-positive/PI-negative), Q3 = late stage of apoptosis (Annexin V- and PI-positive) and Q4 = necrosis (Annexin V-negative/PI-positive). Data represent the mean ± SD, One-way ANOVA followed by Tukey's multiple comparisons test, *p < 0.05; ***p < 0.001 and ****p < 0.0001.
